# ADVERSE CHILDHOOD EXPERIENCES AND LATER LIFE COGNITION: THE MISSING COMPONENT OF MORAL DEVELOPMENT

**DOI:** 10.1093/geroni/igae098.2096

**Published:** 2024-12-31

**Authors:** Rachel Missell-Gray, Alexander Eustice-Corwin, Silvia Sörensen

**Affiliations:** University of Rochester, Rochester, New York, United States; University of Rochester, Rochester, New York, United States; University of Rochester, Warner School of Education and Human Development, Rochester, New York, United States

## Abstract

Recent empirical studies on the relationship between Adverse Childhood Experiences (ACEs) and later life cognition have found an inconsistent association between these two variables. Some studies have determined that a higher number of ACEs are associated with decreased cognitive performance later in life (ages 50 and up), while others have determined that there is no relation. Despite these ambiguous findings, both theory and empirical research provide us with reasons to believe that a relationship between ACEs and later life moral cognition is likely. In the current paper, we argue that self-reported ACEs likely impact older adult moral identity and develop a theoretical framework to guide directions for future research. First, we will summarize the findings of the empirical literature on ACEs and later life cognition. Second, we will address the limitations of these findings. Third, we will advocate in favor of a neo-Aristotelian framework for the study of moral development, which foregrounds the role of practical wisdom (phronēsis) as an alternate conception of a person’s character over the course of the human lifespan. We also argue that such hardships in early life may lead to increased wisdom, which does not necessarily correlate with the measures of cognitive functioning often applied in late-life cognition studies. We hypothesize that the presence of ACEs may influence the development of practical wisdom. We argue that if ACEs predict decreased cognitive function later in life, then this provides us with the grounds for thinking that ACEs might influence moral development in older adults.

